# Evaluation of Myocardial Strain Using Cardiac Magnetic Resonance in Patients with Wilson’s Disease

**DOI:** 10.3390/jcm10020335

**Published:** 2021-01-18

**Authors:** Kun Zhang, Ulrike Reuner, Charlotte Hempel, Uwe Speiser, Karim Ibrahim, Frank R. Heinzel, Burkert Pieske, Marian Christoph, Felix M. Heidrich, Silvio Quick

**Affiliations:** 1 Department of Internal Medicine and Cardiology, Charité—Universitätsmedizin Berlin, Campus Virchow-Klinikum, 13353 Berlin, Germany; frank.heinzel@charite.de (F.R.H.); bukert.pieske@charite.de (B.P.); 2 Berlin Institute of Health (BIH), 10178 Berlin, Germany; 3 DZHK (German Centre for Cardiovascular Research), Partner Site Berlin, 10785 Berlin, Germany; 4 Department of Neurology, Technische Universität Dresden, University Hospital, 01307 Dresden, Germany; ulrike.reuner@tu-dreden.de; 5 Department of Internal Medicine and Cardiology, Technische Universität Dresden, Herzzentrum Dresden Universitätsklinik, 01307 Dresden, Germany; charlotte.hempel@mailbox.tu-dresden.de (C.H.); uwe.speiser@mailbox.tu-dresden.de (U.S.); felix.heidrich@mailbox.tu-dresden.de (F.M.H.); 6 Department of Cardiology, Technische Universität Dresden, Klinikum Chemnitz gGmbH, 09116 Chemnitz, Germany; karim.ibrahim@mailbox.tu-dresden.de (K.I.); marian.christoph@mailbox.tu-dresden.de (M.C.); silvio.quick@mailbox.tu-dresden.de (S.Q.)

**Keywords:** Wilson’s disease, cardiac magnetic resonance imaging, strain analysis, feature-tracking, global longitudinal strain

## Abstract

(1) Background: Wilson’s disease (WD) is an inherited autosomal recessive disorder with the excessive deposition of copper into different organs, including the heart. Previous studies showed structural cardiac changes even in patients with no signs of heart failure. The aim of this study was to perform cardiac magnetic resonance-based strain analysis in WD patients, as it is a powerful independent predictor of mortality. (2) Methods: We conducted a prospective cardiac magnetic resonance study that included 61 patients and 61 age and sex-matched controls, and performed strain analysis of the left and right ventricle. (3) Results: Left ventricular global longitudinal strain (GLS) as a prognostic marker of increased mortality was not altered (control −22.8 (4.8) % vs. WD patients −21.8 (5.1) %, *p* = 0.124). However, 4 of the 61 patients had a markedly reduced GLS. Global circumferential strain did not significantly differ between the groups either (*p* = 0.534). WD patients had significantly reduced global radial strain (*p* = 0.002). Right ventricular GLS was also significantly reduced in WD patients (*p* = 0.01). (4) Conclusions: Strain analysis revealed functional impairment of the left and right ventricle in a small number of patients as a potential early sign of cardiac manifestation in asymptomatic WD patients.

## 1. Introduction

Wilson’s disease (WD) is an inherited autosomal recessive disorder with a prevalence of approximately 1:30,000 and is caused by mutations of the ATP7B gene on chromosome 13. WD leads to reduced copper excretion and excessive deposition of copper into different organs, including the liver, the central nervous system, the cornea, the kidney, the joints, and the heart [1,2,3].

Previous studies, focusing on electrocardiographic changes and standard echocardiographic parameters, showed a mild, non-pathological increase in the thickness of the myocardium and subclinical changes in diastolic function, which has generally been recognized as benign [4,5]. However, evidence of malignancy is accumulating. Cases of cardiomyopathy and lethal arrhythmias have been published [6,7,8]. Recently, we described that signs for structural changes are even present in asymptomatic WD patients who still have normal left and right ventricular dimensions and systolic function in echocardiographic assessment. WD was associated with a higher incidence of premature ventricular ectopic beats (PVB) and severity of WD based on the Unified Wilson’s Disease Rating Scale was significantly correlated to NT-pro BNP [9]. Cardiac magnetic resonance imaging (CMR) unmasked an increase in late gadolinium enhancement (LGE) and a decrease in the right ventricular ejection fraction in WD patients compared to the control [10]. However, the clinical impact and prognostic meaning of these findings are not understood.

Strain analysis in CMR is a new promising tool to capture subtle alterations that result from early diseases of the myocardium, when other parameters such as the ejection fraction are still unaffected by disease. Even though it is a relatively rapid procedure, it is not yet part of routine CMR protocols in clinical practice. In recent studies, CMR based strain analysis has been shown to be a valuable marker for risk prediction. Global longitudinal strain (GLS) was associated with increased all-cause mortality as well as increased death in patients with dilated cardiomyopathy [11,12]. Moreover, Romano et al. showed, in a large multicenter study, that GLS derived from CMR is a powerful independent predictor of mortality in patients with an even preserved ejection fraction [13].

The aim of the present study was to perform strain analysis in WD patients in CMR for the first time. We examined the left and right ventricular functions with strain analysis to detect subtle early myocardial functional changes. As this study was not designed to provide outcome data, we want to discuss our results in light of the current literature and gather information on the risk of heart failure in WD patients, as shown by Grandis et al. [8].

## 2. Experimental Section

### 2.1. Study Design

We performed a prospective imaging study and consecutively enrolled 61 patients with WD that have been transferred to the department of cardiology for cardiac evaluation from 2016 to 2017. WD was reviewed according to the scoring system provided by the 8th International Meeting on WD and Menkes disease. Patients were part of the CARMA Wilson’s disease study (Cardiac manifestation of Wilson’s disease). Conventional CMR data, echocardiography and autonomic function data were published elsewhere [9,10].

We selected 61 age and sex-matched controls from our database with no blood relationship to the patients from our study. Cardiac disease was ruled out in control patients based on the results from clinical examination, echocardiography and CMR. Written informed consent was obtained from all participants. The study protocol conforms to the ethical guidelines of the 1975 Declaration of Helsinki. This study was approved by the local ethics committee (#EK408092015).

### 2.2. Cardiac Magnetic Resonance Imaging and Strain Analysis

We worked with a 3.0 Tesla magnetic resonance system (Signa HDxt 3.0 T, General Electric Company, Milwaukee, WI, USA) using an eight-channel cardiac coil and prospective electrocardiographic wave triggering. Real-time scout images in axial, sagittal, and coronal planes were used to localize the cardiac position within the thorax. From the ventricular apex to the base, ECG-triggered, breath-hold, balanced steady-state free precession sequences (SSFP) were obtained in the short axis, 2-chamber, and 4-chamber view to display the cardiac function. Analysis of the left ventricular global radial, longitudinal, and circumferential, as well as the right ventricular global longitudinal 2D strain values, were obtained using a Feature Tracking Software (TomTec Imaging Systems, Chicago, IL, USA), as previously described [14]. Ventricular volume and function analysis were undertaken by a cardiac post-processing software (Report Card 4.0, General Electric Company, Boston, MA, USA). Calculation of right ventricular (RV) volumes and ejection fraction (EF) was done by manual tracing of endocardial borders of only contiguous short-axis slices at end diastole (first cine phase of the R-wave triggered acquisition) and end systole (image phase with smallest cavity area) without including longitudinal shortening. All patients underwent a late gadolinium enhancement imaging protocol using a segmented inversion-recovery pulse sequence starting 10 to 15 min after a weight-based injection (cumulative dose 0.15 mmol/kg) of gadolinium diethylenetriamine pentaacetic acid (Magnevist, Bayer HealthCare Pharmaceuticals Inc., Berlin, Germany). Regional fibrosis was identified by LGE within the myocardium, defined quantitatively by myocardial postcontrast signal intensity 2 SDs above that within a reference region of remote myocardium within the same slice. Extent of LGE was calculated after manual tracing of endocardial and epicardial borders on each short-axis slice. LGE volume was expressed as a percentage of total myocardial mass (% LGE).

### 2.3. 24 h Electrocardiography

An ambulatory electrocardiograph (ECG, CardioMem^®^ CM 3000, Getemed, Teltow, Germany) was used to detect ventricular premature beats (PVB), and disturbance of rhythm (arrhythmia, tachycardia, and bradycardia). The patients had to wear the ambulatory electrocardiograph for 24 h, while continuing their daily activities.

### 2.4. Laboratory Examination

As cardiac biomarkers we evaluated levels of total creatine kinase (CK, µmol/(sxL)), CK-MB (µmol/(sxL)), myoglobin (µg/L), high sensitive troponin T (hsTroponin T, ng/L) and N-terminal prohormone of brain natriuretic peptide (NT-proBNP, pmol/L).

### 2.5. Statistical Analysis

SPSS 20.0 (SPSS, Inc., Chicago, IL, USA) was used for statistical analyses. Data are presented as mean (SD), median (IQR), or *n* (%) unless otherwise stated. For group comparisons, independent samples t-test and Wilcoxon signed-rank test were used. Pearson’s coefficient was used for correlation analysis. A *p*-value < 0.05 was considered as statistically significant.

## 3. Results

### 3.1. Baseline Characteristics

An overview of the baseline demographic and clinical data of the study population is given in Table 1. The mean age was 44 years for controls and 46 years for WD patients, evenly distributed between male and female. None of the patients had coronary artery disease, symptoms of heart failure or cardiac medication. Detailed clinical characteristics of WD patients are shown in Table 2.

### 3.2. Left Ventricular Strain

Left ventricular global longitudinal strain (GLS) and global circumferential strain did not significantly differ between the groups. WD patients significantly reduced the global radial strain (Table 3). Notably, of the patients, 4 out of 61 showed an average GLS of >−16%.

Patients with GLS > −16% did not significantly differ from patients with GLS < −16% regarding clinical data (patient age in years: 45.3 (9.9) vs. 42.6 (15.4), duration of illness in years: 32.5 (9.95) vs. 22.8 (13.9)), the extent of myocardial fibrosis (LGE in %: 1.1 (2.5) vs. 1.4 (1.8)), premature ventricular beats (PVB) per 24 h (57.5 (61) vs. 260.6 (1096)) and NT-proBNP in ρmol/L (4.3 (3.6) vs. 6.8 (10)) as well as all other examined cardiac biomarkers (i.e., CK, CKMB, hsTroponin T).

### 3.3. Right Ventricular Strain and Ejection Fraction

Right ventricular function assessed by global longitudinal strain of the right ventricle was significantly reduced in WD patients (Table 3). Two of the 61 patients had reduced RVEF (<40% limits indicated by the analysis software).

### 3.4. Late Gadolinium Enhancement

The extent of LGE was significantly higher in WD patients compared to controls (4.9 ± 1.4% vs. 1.1 ± 0.2%; *p* = 0.003). LGE was most frequently observed at the right ventricular insertion point (58 of 61 WD patients vs. 3 of 61 controls, *p* < 0.001). Eleven of the 61 WD patients showed a midmyocardial LGE streak in the interventricular septum; none of the controls showed that pattern (*p* < 0.001).

### 3.5. 24 h Electrocardiographic Recording

Heart rhythm disturbances were seen in 4 WD patients (3 patients had an ectopic atrial rhythm, 1 patient had atrial flutter). One patient suffered from a high-grade atrioventricular block, which required permanent pacemaker implantation. Ventricular ectopic beats (VEB) were detected in 30 of 61 WD patients (49%). In average 216.3 VEB (range 0; 6727) were seen. However, we could not detect any potentially fast malignant arrhythmias.

### 3.6. Laboratory Data

Cardiac biomarker levels of WD patients are presented in Table 4. Notably, in none of the patients could elevated NT-pro-BNP levels be detected. High sensitive troponin T above normal range was evident in 4 of the 61 patients, not being those with impaired GLS.

### 3.7. Correlation Analysis

**Figure 1:**
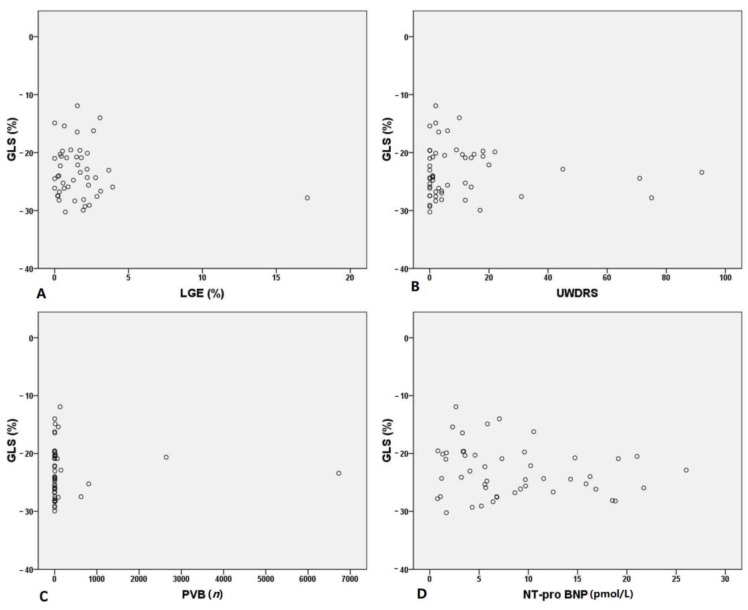
Correlation analysis of global longitudinal strain (GLS) and late gadolinium enhancement (LGE) (**A**), Unified Wilson’s Disease Rating Scale (UWDRS) (**B**), premature ventricular beats (PVB) (**C**) and NT-pro BNP (**D**).

We performed correlation analysis using GLS, which has been described as a powerful independent risk predictor of mortality. Overall, GLS did not correlate with LGE (*p* = 0.353), Unified Wilson’s Disease Rating Scale (*p* = 0.666), PVB (*p* = 0.934) or NT-pro BNP (*p* = 0.223) (Figure 1). Moreover, no correlation was found between the incidence of a GLS > −16% (4 out of 61 patients) and these parameters.

## 4. Discussion

This is the first CMR-based strain analysis study in WD. Moreover, to date, it is the largest prospective imaging study evaluating cardiac manifestation of WD. Our results show that GLS, although in the normal range in the majority of patients, was significantly reduced in a small number of patients. Furthermore, left ventricular GRS and right ventricular GLS were significantly decreased in WD patients compared to healthy controls.

In 1987, cardiac manifestation was described by Kuan et al. [7]. Based on observations in 53 patients, he concluded that cardiac involvement in Wilson’s disease includes arrhythmias, cardiomyopathy, cardiac death, and autonomic dysfunction. Since then, several studies reported on a broad variety of abnormal electrocardiographic findings. However, structural changes are rarely seen in the usually stable WD patients under medical treatment, when transferred to standard cardiac examination [4]. As presented previously, asymptomatic WD patients had grossly normal systolic left heart (ejection fraction, end diastolic diameter) and right heart function (end diastolic diameter, tricuspid annular plane systolic excursion, right ventricular systolic pressure) in echocardiography; only mild diastolic dysfunction was present compared to healthy controls [9].

To further evaluate the subtle cardiac changes in our group of clinically asymptomatic patients, we used the new method of CMR-based strain analysis, also referred to as “feature-tracking” [15] in the present study. It provides detailed information on global and regional active LV deformation. Subtle changes in the structure and geometry of the LV myocardium may lead to changes in LV deformation that may not be detectable with LV ejection fraction. Especially the determination of GLS is of practical value, because a worsening of GLS has been associated with a higher mortality, even in patients with preserved ejection fraction [11,12,13,16,17,18,19]. We could show that GLS was not altered in WD patients compared to controls. However, in 4 out of 61 patients, a GLS > −16% was observed. In a recent published study by Froöjdh et al. of consecutive adult patients, who were referred to their center for standard CMR, a GLS > −16% was associated with a higher mortality [20]. Moreover, from a previously published longitudinal cohort study we know that WD patients carry a higher risk of heart failure (HF), even after adjustment for potential confounders and mediators like hypertension, diabetes mellitus, and coronary artery disease [8]. Due to limitations in the study design lacking cardiac imaging data, the authors could only speculate about an underlying cause. The reduced myocardial GLS in a small proportion of our WD patients could be interpreted as a subtle precursor of developing heart failure, but still does not resolve the question of causality. Regarding clinical data (age, duration of illness), medication use, cardiac biomarkers and myocardial fibrosis, we did not find any obvious cause for the reduction of myocardial strain in these patients. A direct cardiotoxic effect of copper on the myocardium can be discussed [21,22]. We conclude that deformation measurements such as GLS, reflecting subtle cardiomyocyte contractile dysfunction, may serve as cardiac risk predictors, albeit causality in WD patients is not yet known.

Interestingly, we detected a reduction in global radial strain. A reasonable explanation could be hypertrophy and the increase in LV mass that can be observed in WD patients [10,23], since a correlation between hypertrophy and radial strain has been shown before [24].

Previous studies have not focused on right ventricular function in WD patients. We demonstrate that WD patients show a reduced RV systolic function, which is reflected in significantly impaired RVGLS, RVEF and RVFAC compared to control subjects. This is of clinical relevance because right ventricular function is a strong independent predictor of short and long-term mortality in many different conditions [25,26].

Whether GLS is superior in detecting early myocardial damage remains a matter of investigation, as this study does not include outcome data. Furthermore, previous studies showed a good correlation of RVGLS and RVEF [27,28]. Advantageously, however, GLS can also be determined by echocardiography, whereas the complex anatomy and physiology of the right ventricle is often a major limitation of RVEF measurement [29].

In a recent echocardiography study, Gavazzoni et al. showed that the GLS of the right ventricular free wall is independently associated with the outcome [30]. The prognostic value of right ventricular GLS derived by CMR is not established so far and the subject of current studies.

## 5. Conclusions

Strain analysis revealed functional impairment of the left and right ventricle in a small number of patients as a potential early sign of cardiac manifestation in asymptomatic WD patients. The cause of GLS deterioration remains unclear as it does not correlate with the degree of fibrosis. The clinical significance of these findings regarding outcome parameters has to be evaluated in long-term studies.

## 6. Limitations

Markedly reduced strain parameters of the left and right ventricle were only evident in a small number of patients. This might firstly be due to the fact that the majority of our cohort, mostly stabilized symptomatic patients or asymptomatic subjects, were well-treated. Secondly, our cohort was on average more than 20 years younger than in a previous study that could detect a higher risk of heart failure in WD patients. Probably, older patients with higher disease severity or during acute exacerbation would even show more marked signs of structural heart disease, as determined by myocardial strain imaging.

To gain more insight, a larger study population with a broader range of disease severity would be desirable for future studies.

## Figures and Tables

**Table 1:** Demographic and clinical parameters of the study population.

	WD Patients (*n* = 61)	Controls (*n* = 61)
Age (years)	46 ± 14	44 ± 15
Male	31 (51)	31 (51)
BMI (kg/m^2^)	26 ± 7.0	24.9 ± 3.8
Arterial Hypertension	12 (19.7)	9 (14.8)
Diabetes mellitus	0 (0)	1 (1.6)
Hyperlipoproteinemia	6 (9.8)	4 (6.6)
Obesity	2 (3.3)	1 (1.6)
Coronary artery disease	0 (0)	0 (0)
Symptoms of heart failure	0 (0)	0 (0)

Data are presented as mean (SD) or *n* (%) unless otherwise stated.

**Table 2:** Clinical characteristics of WD patients.

Variable	Data (*n* = 61)
Duration of disease in years	24.9 (14.7)
Phenotypic presentation at the time of diagnosis ^8^	
Hepatic ^1^	27 (44.3%)
Neurologic/psychiatric with symptomatic liver disease	7 (11.5%)
Neurologic/psychiatric without symptomatic liver disease	18 (29.5%)
Asymptomatic ^2,3^	9 (14.8%)
Phenotypic presentation at the time of investigation ^8^	
Hepatic ^1^	27 (44.3%)
Neurologic/psychiatric with symptomatic liver disease	21 (34.4%)
Neurologic/psychiatric without symptomatic liver disease	4 (6.6%)
Asymptomatic ^2,3^	9 (14.8%)
Patients with acute exacerbation within the disease process ^4^	18 (29.5%)
Patients after liver transplantation	4 (6.6%)
Liver fibrosis according to METAVIR score ^5^	
F0	27 (44.3%)
F1	7 (11.5%)
F2	4 (6.6%)
F3	8 (13.1%)
F4	15 (24.6%)
lPatients with pathological cranial MRI ^6^	32 (52.5%)
Therapy	
Penicillamine	37 (60.7%)
Trientine	13 (21.3%)
Zinc	7 (11.5%)
No Wilson’s disease medication ^7^	4 (6.6%)

Data are presented as mean (SD) or *n* (%) unless otherwise stated. ^1^ Symptomatic liver disease or asymptomatic elevation of liver enzymes with exclusion of neurological manifestation. ^2^ Genetic testing within family screening. ^3^ No evidence of hepatic or neurologic symptoms. ^4^ Wilson’s disease dependent impairment of health condition which leads to hospitalization. ^5^ Liver fibrosis according to Metavir score using Data of transient Elastography (Fibroscan^®^). ^6^ Evidence of characteristic pathological changes in the cranial MRI. ^7^ Patients after liver transplantation. ^8^ Modified by Ferenci.

**Table 3:** Myocardial strain and CMR characteristics of patients and controls.

	WD Patients (*n* = 61)	Controls (*n* = 61)	*p*-Value
Left ventricular parameters			
GLS, %	−22.8 (4.8)	−21.8 (5.1)	0.124
GRS, %	43.2 (13.2)	51.6 (13.8)	0.002
GCS, %	−29.2 (5.2)	−28.6 (4.7)	0.534
LVEF, %	66.1 (5.0)	65.2 (2.7)	0.382
LVEDV, mL	123.7 (38.8)	139.0 (35.5)	0.004
LV mass, g	114.0 (31.0)	104.0 (34.0)	0.003
Right ventricular parameters			
GLS, %	−23.6 (4.9)	−26.1 (5)	0.01
RVEF, %	45.7 (3.0)	49.4 (7.6)	<0.001
RVFAC, %	51.7 (4.2)	53.6 (3.6)	0.018
RVEDV, mL	125.3 (38.0)	122.5 (36.3)	0.614

Data are presented as mean (SD). GLS—global longitudinal strain; GRS—global radial strain; GCS—global circumferential strain; LVEF—left ventricular ejection fraction; LVEDV—left ventricular end-diastolic volume; RVEF—right ventricular ejection fraction; RVFAC—right ventricular fractional area change; RVEDV—right ventricular end-diastolic volume.

**Table 4:** Cardiac biomarker levels of Wilson’s disease patients.

Cardiac Biomarker	Data (*n* = 61)	Within Normal Range	Pathological Values
Total CK, µmol/(sxL)	1.7 (1.0)	52 (85%)	9 (15%)
CK-MB, µmol/(sxL)	0.3 (0.1)	55 (90%)	6 (10%)
Myoglobin, µg/L	30.9 (24.0)	53 (87%)	8 (13%)
hsTroponin T, ng/L	4.0 (2.0)	57 (93%)	4 (7%)
NT-proBNP, ρmol/L	8.6 (6.8)	61 (100%)	-

Data are presented as mean (SD) or *n* (%). CK—creatine kinase; NT-proBNP—N-terminal prohormone of brain natriuretic peptide; hsTroponin T—high sensitive Troponin T.

## Data Availability

The data presented in this study are available on request from the corresponding author. The data are not publicly available due to ethical restrictions.
